# Hydric stress-dependent effects of *Plasmodium falciparum *infection on the survival of wild-caught *Anopheles gambiae *female mosquitoes

**DOI:** 10.1186/1475-2875-9-243

**Published:** 2010-08-26

**Authors:** Fred Aboagye-Antwi, Amadou Guindo, Amadou S Traoré, Hilary Hurd, Mamadou Coulibaly, Sékou Traoré, Frédéric Tripet

**Affiliations:** 1 Center for Applied Entomology and Parasitology, School of Life Sciences, Keele University, Keele, Staffordshire ST5 5BG, UK; 2 Malaria Research and Training Center, Faculty of Medicine and Dentistry, University of Mali, Bamako, Mali

## Abstract

**Background:**

Whether *Plasmodium falciparum*, the agent of human malaria responsible for over a million deaths per year, causes fitness costs in its mosquito vectors is a burning question that has not yet been adequately resolved. Understanding the evolutionary forces responsible for the maintenance of susceptibility and refractory alleles in natural mosquito populations is critical for understanding malaria transmission dynamics.

**Methods:**

In natural mosquito populations, *Plasmodium *fitness costs may only be expressed in combination with other environmental stress factors hence this hypothesis was tested experimentally. Wild-caught blood-fed *Anopheles gambiae *s.s. females of the M and S molecular form from an area endemic for malaria in Mali, West Africa, were brought to the laboratory and submitted to a 7-day period of mild hydric stress or kept with water ad-libitum. At the end of this experiment all females were submitted to intense desiccation until death. The survival of all females throughout both stress episodes, as well as their body size and infection status was recorded. The importance of stress, body size and molecular form on infection prevalence and female survival was investigated using Logistic Regression and Proportional-Hazard analysis.

**Results:**

Females subjected to mild stress exhibited patterns of survival and prevalence of infection compatible with increased parasite-induced mortality compared to non-stressed females. Fitness costs seemed to be linked to ookinetes and early oocyst development but not the presence of sporozoites. In addition, when females were subjected to intense desiccation stress, those carrying oocysts exhibited drastically reduced survival but those carrying sporozoites were unaffected. No significant differences in prevalence of infection and infection-induced mortality were found between the M and S molecular forms of *Anopheles gambiae*.

**Conclusions:**

Because these results suggest that infected mosquitoes may incur fitness costs under natural-like conditions, they are particularly relevant to vector control strategies aiming at boosting naturally occurring refractoriness or spreading natural or foreign genes for refractoriness using genetic drive systems in vector populations.

## Background

Determining whether *Plasmodium falciparum*, the agent of human malaria responsible for over a million deaths per year, causes fitness costs to its mosquito vector *Anopheles gambiae *has critical implications for the understanding of mosquito/malaria interactions. There is mounting evidence that susceptibility to the parasite may be determined by allelic variation at a limited number of loci [1,2]. Unravelling the evolutionary forces that determine the frequency of such susceptibility and refractory alleles is important for understanding the evolutionary ecology of this, and other, vector-parasite associations and will feed back into the general understanding of the dynamics of malaria transmission, which crucially depends on vector-parasite interactions [3]. In addition, such advances could help scientists devise new ways to boost naturally occurring refractoriness in vector populations. Finally, such knowledge is also important for vector control projects aiming to spread natural or foreign genes for refractoriness using genetic drive systems [4]. Currently, there are still doubts about the occurrence of *Plasmodium*-imposed fitness costs on its vector host and how those fitness costs are modulated [5-7].

The high frequency of refractoriness alleles found in natural populations implies that *P. falciparum *are damaging to mosquito hosts and that an immune response can minimize these costs [1,2]. However, mounting an immune response can itself be costly and could explain the maintenance of susceptibility alleles, particularly when parasite prevalence in mosquitoes is low or varies seasonally [7]. A number of studies have attempted to detect fitness costs of malaria on mosquitoes using non-natural mosquito/*Plasmodium *associations. For example, reduced fecundity was shown in *Aedes aegypti *infected with *Plasmodium gallinaceum *[8,9] and anopheline mosquitoes infected with *Plasmodium yoelii nigeriensis *[10,11]. Unfortunately, results from these laboratory-based model systems cannot be extrapolated to natural mosquito/*Plasmodium *populations because of unrealistic parasite loads and the lack of coevolutionary history between their vector and *Plasmodium *components (discussed in [12]). However, a reduction in fecundity was also found in *Anopheles gambiae *s.l. naturally infected with *Plasmodium falciparum *in Tanzania in one of the rare field-based study of fitness costs [13].

Reduced survival of female mosquitoes has sometimes also been associated with infection but Ferguson and Read [5] showed in a meta-analysis of published studies that those effects were only significant when considering non-natural, laboratory-based host-parasite associations. The rare studies that have looked at survival costs of *P. falciparum *in its natural *An. gambiae *host have found mixed results [14,15]. Anderson *et al *[15] reported an increase in feeding persistence in infected *An. gambiae *females, which resulted in a higher proportion getting killed while feeding on human hosts. However, so far no direct negative effects of the *Plasmodium *infection itself were found on mosquito survival [14].

In field populations, host-parasite interactions are modulated by genetic and environmental factors. At the individual level within a population, the fitness of an infected host and its immune response depend on that individual's genetic background and the reaction of that genetic background with past and current environmental factors that determine its body condition during the course of an infection. The ensemble of all individuals' reaction norms constitutes the whole range of phenotypic variation observed in natural host populations. So far, the importance of genetic and environmental factors on the fitness of *An. gambiae *infected with *P. falciparum *has only been tested in the laboratory where both mosquito and parasite exhibit limited variation in genetic background and environmental conditions [7,12]. Thus, in order to understand the evolutionary forces that maintain variation in susceptibility and resistance to *Plasmodium *infections in wild mosquito populations, it is paramount to examine fitness costs in relation to environmental conditions that better match those occurring in the natural setting.

One such environmental condition is limited adult access to water. In the laboratory, mosquitoes are constantly supplied with water often combined with sugar as sugar-water. Whilst these conditions might arguably match those found in tropical humid or equatorial regions of Africa, in semi-arid regions of sub-Saharan Africa water is not often found near mosquito resting sites and seasonal variations in water availability and ambient humidity are important determinants of mosquito abundance and distribution [16,17]. As an example, in Mali, the relative abundance of the Mopti, Savanna and Bamako chromosomal forms of *An. gambiae *is strongly associated with seasonal rainfalls thereby underlining the role of certain chromosomal inversions in conferring resistance to desiccation [18]. The Mopti chromosomal form, which is the most resistant to drought, predominates in dry areas and during the dry season [19,20]. In contrast, the Bamako and Savannah forms dominate in wetter parts of Mali and their abundance and geographical range increases with the rainy season [19,20]. Thus drought and the selection pressures associated with hydric stress (stress induced by limited water availability) play a critical role in the evolution and maintenance of genetic polymorphism in *An. gambiae'*s populations and are important determinants of the chromosomal forms' distribution and abundance [19,21]. Aside from seasonal droughts, mosquito populations in sub-Saharan Africa are also subjected to large daily fluctuations in temperature and relative humidity. Consequently, adaptations for coping with hydric stress and maintaining body water balance are considered some of the most important aspects of mosquito behaviour and physiology [22].

The effect of hydric stress was tested experimentally in naturally-infected and uninfected *An. gambiae *s.s. females collected from huts in the surroundings of Bancoumana a small locality in Mali, West Africa. This region is hyper-endemic for malaria and its vector populations are dominated by *An. gambiae *s.s. [20]. Both M and S ribosomal DNA molecular forms are present in the area, with the M form featuring inversion polymorphisms typical of the drought-tolerant Mopti chromosomal form [19], whilst the S molecular form shows chromosomal arrangements typical of the Savanna and Bamako forms [20]. Blood-fed *An. gambiae *females were collected and assigned to two experimental groups. In the first one, they were given full access to water to simulate laboratory-like conditions; in the second one, limited access to water to simulate natural-like conditions wherein access to humidity may be regulated by mosquito behaviour but water is often unavailable for long periods of time. Female survival was then followed and, when they died, their infection status, body size, and molecular form were determined. Following the experimental hydric stress period, all surviving females were submitted to a desiccation challenge until dead, a procedure used in physiological studies to reveal underlying differences in metabolic reserves [22,23], and which was used here to best simulate a life-threatening desiccation episode in nature.

The results of this study provide indirect and direct evidence suggesting that infection with *Plasmodium *interacts with hydric stress to impose fitness costs on *An. gambiae *females in terms of survival. These costs were expressed in females with limited access to water and equally affected the M and S molecular form individuals. This is the first study suggesting a significant negative effect of infection on *An. gambiae *female mosquito survival due to the direct impact of *Plasmodium *infection.

## Methods

### Study sites and mosquito collection

Resting catches of blood-fed *An. gambiae *s.l. females were carried out in the early morning from huts in six compounds in the villages of Kenieroba (8°, 33' W and 12°, 11' N) and Fourda (8°, 34' W and 12°, 09' N). Both are fishing and farming communities located 71 km southwest of the capital of Mali, Bamako. The two villages are about 2 km apart, share the same mosquito population, malaria transmission dynamics and climatic conditions. The collections were done for three days in mid October 2008, towards the end of the rainy season. Resting blood-fed females were collected with mouth aspirators and stored in paper cups under a humid towel until they were brought back to the insectary of the Malaria Research and Training Center (MRTC) and distributed in four one-gallon cardboard cages with access to sugar-water. Insectary conditions were a temperature of 25°C, 80% relative humidity (RH) and 12:12 h light-darkness cycles.

### Experimental design

#### General scheme

Field collected blood-fed females were distributed over two experimental groups, the first one were given full access to water for 7 days, the second were submitted to hydric stress by having restricted access to water for 7 days. At the end of that period females from both experimental groups were submitted to continuous dry conditions or desiccation challenge until dead.

### Detailed procedures

Following the field collections, mosquitoes were morphologically determined using the criteria described in the identification keys of Gillies and de Meillon [24] and Gillies and Coetzee [25] and 480 *Anopheles gambiae *s.l. females were distributed from the four cages into 18 pots (25-30 individuals per pot). To ensure that females were randomly distributed among pots, the cages were regularly shaken and females collected from alternate heights in the cage. The 18 pots were sequentially assigned to two experimental treatments thereby controlling for any remaining bias in female phenotype. All females were provided with sugar in the form of sugar cubes. In the first experimental group females were provided with water-impregnated cotton pads 24 h/day (non-stressed group) but in the second group the water-impregnated cotton pads were removed for eight hours during the daytime resulting in females having access to water for 16 h/day only (hydric-stressed females). Previous experiments comparing survival in both groups showed that such reduction in water availability did not differentially affect stressed females (Proportional Hazards likelihood ratio: *P *< 0.4 in both cases, see discussion). This treatment regimen was continued until day 7 after commencement of the experiment and constituted what is referred to as '7-day stress period' phase of the experiment. No oviposition pots were provided during that period. Special care was taken to alternate the position of the pots on the shelves in the insectary so as to avoid confounding factors due to their physical location. During this phase of the experiment, the pots were monitored for dead females (those unable to stand were considered dead) at 4 h intervals. Each dead mosquito was recorded, collected and split into three parts under the binocular microscope. The wings were glued onto microscope slides for wing length measurements at a later stage and the abdomens stored in PBS at -20°C for dissections of the mid-gut for detection of *P. falciparum *oocysts at the end of the experiments. The head and thorax of each mosquito was placed in a 1.5 ml micro centrifuge tube and homogenised in 50Μl BBNP40 (Nonidetp-40) with pestles. The pestles were then rinsed with 200Μl BB (ELISA blocking buffer) into the tube making up a total volume of 250Μl. Samples were then frozen at -20°C for later assessment of sporozoite infections by ELISA and mosquito species diagnosis by PCR.

On day 7 of the experiment, all mosquitoes that survived the 7-day hydric stress period were deprived of water and sugar, and subjected to a strong desiccation stress by placing them in an incubator at 30°C and 30% RH. This is a life-threatening challenge that creates conditions of extreme drought with which mosquitoes can cope only for a limited amount of time. This phase of the experiment is referred to as 'desiccation challenge' phase of the experiment. Hourly mortality was recorded and each dead mosquito processed as described above for the 7-day hydric stress phase that preceded the desiccation challenge.

### Parasite prevalence in relation to hydric stress

Mid-gut dissections were conducted on all females that died during the course of the experiment to detect one or more oocysts originating from feeding on an infected human the night before their collection, or mature oocysts from an older infection. Mid-guts of individual mosquitoes were dissected in phosphate buffered saline (PBS, pH 7.2), stained in 0.01% mercurochrome and examined under light microscope at 400× magnification. Invading ookinetes and very young oocysts cannot be detected using this method (day 0 to day 4 of the experiment) and they are also particularly hard to detect by PCR method because of contaminating parasite DNA in the bloodmeal. Consequently, new infections in females that died early (i.e. younger than 4 days-old) could not be directly detected and these females were involuntarily recorded as non-infected resulting in an underestimation of oocyst rates and preventing a direct assessment of early parasite-induced mortality. To circumvent that problem, parasite-induced mortality occurring at the early stage in relation to hydric stress was inferred indirectly from: (1) Comparing patterns of mortality in both groups of females in the early stages of the experiment and (2) Comparing the frequency of infection detected in both groups at a later stage in the experiment. If parasite induced-mortality increased under stress in the early stages of parasite development, this would result in an overall decrease of oocysts infections detected in surviving females at a later stage. This predicted pattern of mortality and infection prevalence distinguishes the potential effects of hydric stress on parasite-induced fitness costs from other interpretations and was used as the working hypothesis (see Table 1).

**Table 1:** Potential impact of hydric stress on female survival and infection prevalence during 7-day stress period

Potential impact of hydric stress			Predicted measured effects	
**Hypothesis**	**Direct negative** **effect on survival**	**Increased parasite-induced** **mortality**	**Female** **survival**	**Infection** **prevalence**

1	No	No	none	none
2	Yes	No	decreased	none
**3**	**No**	**In early stages of infection (days 0-4)**	**decreased(days 0-4)**	**decreased**
4	Yes	In early stages of infection (days 0-4)	decreased(days 0-7)	decreased
5	No	In late stages of infection (days 4-7)	decreased(days 4-7)	none
6	Yes	In late stages of infection (days 4-7)	decreased(days 4-7)	none

Mortality incurred through the direct effect of hydric stress is distinguished from that due to increased parasite-induced mortality in females under stress. The occurrence of one or both of these sources of mortality and their timing leads to several possible hypotheses (1-6), each yielding distinct predicted patterns of measured female survival and overall oocyst prevalence. Because ookinetes and very young oocysts in early-dying females (days 0-4) are undetectable, an increase in parasite-induced female mortality in response to hydric stress at that stage translates in an apparent decrease in the prevalence of oocysts detected in females dying later in that experimental group (hypothesis 3 - in bold).

Sporozoite detection by enzyme linked immunosorbent assay (ELISA) were conducted on all females dying during the experiment in order to detect sporozoites from previous infections, as well as sporozoites that developed during the course of the experiment in females which already had oocysts when they were collected. Fifty microliters of each mosquito homogenate were distributed into an anti-*Plasmodium falciparum *sporozoite monoclonal antibody (Mab) coated 96-well plate following the method developed by Burkot *et al *[26] and Wirtz *et al *[27] based on the detection of circumsporozoite (CS) antigen. Plates were incubated for 2 h at room temperature. The homogenates were then removed; the wells washed, and captured CS antigens revealed by incubation with a Mab-peroxidase conjugate for 1 h. After incubation, the conjugate was removed, the wells washed again and 100Μl of ABTS substrate was added to each well, followed by another 30 min of incubation. Plates were then read visually with wells clearly coloured green corresponding to sporozoite-positive female mosquitoes. Such assays have been used routinely and for several years at the MRTC for parasite detection in thousands of field-collected mosquitoes. Every assay features positive and negative controls to ensure that potential problems related to the preparation of the assay are detected. Comparisons with other detection methods as well testing of large numbers of uninfected samples have shown that the assay is generally robust and accurate [28-30]. It is thought that it's capacity to detect circumsporozoite proteins shed by migrating sporozoites may overestimate true salivary gland infections compared to salivary gland dissections but that the ELISA is slightly less sensitive than some of the most PCR-based detection methods [31].

### Mosquito ID and size

Genomic DNA was extracted from the homogenate of head and thorax of all *An. gambiae *s.l. individuals using DNAzol (Invitrogen) following the manufacturer's instructions. The PCR-restriction digest procedure developed by Fanello *et al *[32] was used for distinguishing *An. arabiensis *individuals from *An. gambiae s.s*. and within the later sub-taxon, individuals belonging to the M and S molecular forms.

Wing length was used as a good correlate of mosquito body size [33]. The wings of dissected female mosquitoes were measured from the alular notch to the distal wing margin, excluding the fringe scales, to the nearest 0.01 mm using a binocular microscope with an eyepiece graticule.

### Statistical procedures

All statistical analyses were performed using the software JMP7.02 (SAS Institute, Inc). All data were checked for normality and heteroscedasticity and analysed accordingly using parametric or non-parametric procedures. In order to test the effect of hydric stress on oocyst and sporozoite prevalence, the frequency of females in which we detected parasites (dependent variable) was analyzed in relation to the independent variables water availability, molecular form, and body size (measured as wing length) using multivariate logistic regressions. Differences in survival between females in which oocysts and sporozoites were detected were analysed through proportional hazards survival analyses, a multivariate approach which tolerates deviations from normality. In this case female survival (dependent variable) was analysed in relation to the independent variables, water availability, oocyst and sporozoite presence, molecular form, and body size. Non-significant interactions were removed from the multivariate models unless otherwise indicated.

## Results

### Predicted patterns of infection prevalence and female survival

As highlighted in the method section, because early stage infections (day 0-4) cannot be detected by mid-gut dissection, the potential interactive effect of hydric stress and newly contracted infections on female survival was assessed indirectly by comparing female survival in stressed and non-stressed females at different stages of the experiment and by comparing infection prevalence in both groups (Table 1, hypothesis 3). If early infection stages significantly affected the survival of stressed females, the measured prevalence of oocyst infections in stressed female that survived longer than four days was expected to be significantly lower than in non-stressed females because fewer infected stressed females actually survived to develop detectable oocysts. In contrast, sporozoites arose from previous bloodmeals and infections and were assumed to be detectable by ELISA independently of the stage of the experiment at which the mosquito died, hence their prevalence and impact on survival was directly assessed (see result sections below).

### Species composition and sample sizes

Out of the 480 females morphologically identified as *An. gambiae *s.l., 10 were characterized as *An. arabiensis *and 430 as *An. gambiae *s.s. by PCR assay. Of the 40 PCR assays that failed, 24 were females that died before the end of the 7-day stress period (14 from the water-restricted group and 10 from the group with water ad-libitum) and 16 were females that survived the 7-day stress period until the desiccation challenge (seven from the stressed group and nine from the non-stressed group). The PCR-based diagnostic revealed that 238 *An. gambiae s.s *females belonged to the S molecular form and 192 to the M molecular form, equivalent to the Mopti chromosomal form in this region. All analyses were performed on the 430 females identified as *An. gambiae s.s*.

### Parasite prevalence

The mid-gut of 400 females out of 430 was successfully dissected for oocyst presence and all heads and thoraces were tested for sporozoite presence by ELISA. The dissection of female mosquito midguts resulted in the detection of oocysts in 13 females out a total of 400 females, equivalent to a 3.3% prevalence of infection (Table 2). The mean number of oocysts was 1.07 and the median was equivalent to 1. Oocysts were found in 8.5% females that died during the 7 days preceding the desiccation challenge (Table 2). This percentage dropped drastically in the fraction of females that died during the desiccation challenge that followed, with only 1.9% females found carrying oocysts in their midguts (*Chi*-square: *n *= 400, *df *= 1, *χ*^2 ^= 9.17, *P *= 0.003)(Table 2).

**Table 2:** Oocyst and sporozoite prevalences during 7-day hydric stress period and desiccation challenge

Experimental phase	7-day hydric stress			Desiccation challenge			Hydric stress + Desiccation		
**Water availability**	**16 h**	**24 h**	**All**	**16 h**	**24 h**	**All**	**16 h**	**24 h**	**All**

Oocysts (%)	2.4 (1/41)	14.6 (6/41)	8.5 (7/82)	0.6 (1/159)	3.1 (5/159)	1.9 (6/318)	1.0 (2/200)	5.5 (11/200)	3.3 (13/400)
Sporozoites (%)	4.9 (2/41)	9.8 (4/41)	7.3 (6/82)	10.4 (18/173)	14.9 (26/175)	12.6 (44/348)	9.3 (20/214)	13.9 (30/216)	11.6 (50/430)
Both (%)	0 (-/41)	2.4 (1/41)	1.2 (1/82)	0 (-/159)	0.6 (1/159)	0.3 (1/318)	0 (-/200)	1 (2/200)	0.5 (2/400)

Prevalence of oocysts and sporozoites (%), and the number of cases over total sample sizes (in brackets) detected in field-collected *An. gambiae *females that died during the 7-day hydric stress phase of the experiment and during the ensuing desiccation challenge. During the hydric stress period females were kept either with restricted access to water (16 h) or with water *ad-libitum *(24 h).

The reverse of that pattern was observed for sporozoites detected by ELISA. An overall prevalence of 11.6% was measured (Table 2). Sporozoites were found in 7.3% females that died during the 7-day stress period but their frequency increased, although non-significantly so, to 12.6% of females undergoing the desiccation challenge (*Chi*-square: *n *= 430, *df *= 1, *χ*^2 ^= 1.83, *P *= 0.176)(Table 2).

### Hydric stress and prevalence of infection

The effects of hydric stress, molecular form (M or S) and body size (measured as wing length) on the prevalence of oocysts detected in females were examined by multivariate logistic regression. Hydric stress significantly decreased the prevalence of oocysts infection (Table 3) thereby following a pattern of oocyst prevalence compatible with early infection-induced mortality (Table 1 - Hypothesis 3). Two out of 200 (1.0%) females from cages with limited access to water were found with oocysts as compared to 11 out of 200 (5.5%) females from cages with water *ad libitum *(Table 2). The M and S molecular form of *An. gambiae *s.s. did not differ in oocyst prevalence, and no relationship was found between female body size and prevalence of oocyst infection (Table 3).

**Table 3:** Test of effects of water availability, molecular form and body size on oocyst prevalence

Source	*df*	L-R *Chi*-square	*p*-value
Water availability	1	7.12	**0.008**
Molecular form	1	1.50	0.220
Body size	1	0.58	0.446

Nominal logistic regression (Likelihood-ratio tests presented here, *n *= 400) of the effects of water availability, molecular form and body size on the prevalence of oocysts detected in blood-fed *An. gambiae *s.s. female mosquitoes collected from huts in an area endemic for malaria and brought to the laboratory. Females were kept for 7 days with water *ad-libitum *or with restricted access to water before being submitted to constant desiccation until dead. Significant *p*-values are in bold. Interactions were non-significant.

The effects of hydric stress, molecular form (M or S) and body size (measured as wing length) on sporozoite prevalence during the whole course of the experiment were examined by multivariate logistic regression. We found no significant decrease of sporozoite prevalence (Table 4). Sporozoites were found in 20 out of 214 (9.3%) females from cages with limited access to water as compared to 30 out of 216 (13.9%) females from cages with water *ad libitum*. Neither the females' molecular form nor their body size affected sporozoite prevalence (Table 4).

**Table 4:** Test of effects of water availability, molecular form and body size on sporozoite prevalence

Source	*df*	L-R *Chi*-square	*p*-value
Water availability	1	2.08	0.150
Molecular form	1	0.66	0.418
Body size	1	1.06	0.302

Nominal logistic regression (Likelihood-ratio tests presented here, *n *= 430) of the effects of water availability, molecular form and body size on the prevalence of sporozoites in blood-fed *An. gambiae *s.s. female mosquitoes collected from huts in an area endemic for malaria and brought to the laboratory. Females were kept for seven days with water *ad-libitum *or with restricted access to water before being submitted to constant desiccation until dead. Interactions were non-significant.

### Hydric stress, infection and female survival

**Figure 1:**
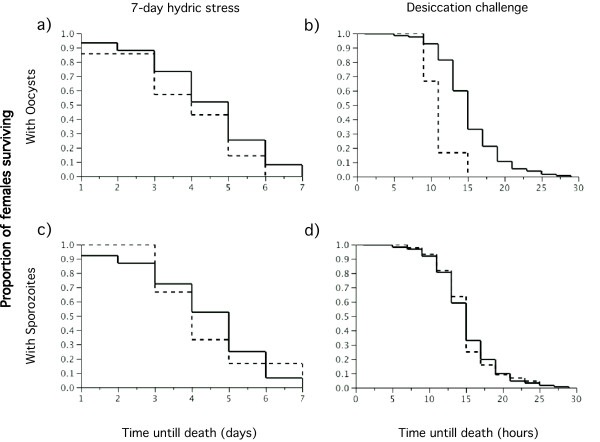
**Survival of *An. gambiae *s.s. females infected with oocysts (a,b dashed line) and sporozoites (c,d dashed line) and those found without parasites (a-d solid lines)**. Survival curves were calculated for the 7-day period with experimental manipulation of water availability (a,c) and for the shorter desiccation challenge (b,d).

The overall effects of hydric stress, molecular form, body size and presence of oocysts or sporozoites on the survival of female mosquitoes during the 7-day hydric stress period and the desiccation challenge were investigated by multivariate proportional hazards analyses. Survival during the 7 days preceding the desiccation challenge was independent of the above variables including the experimental manipulation of hydric stress, and infection with oocysts and sporozoites (Table 5, Figure 1a,c).

**Table 5:** Analysis of determinants of female survival during 7-day stress period

Source	*df*	L-R *Chi*-square	*p*-value
Water availability	1	0.02	0.881
Molecular form	1	0.52	0.473
Body size	1	0.78	0.378
Oocyst(s) presence	1	0.85	0.357
Sporozoites presence	1	0.03	0.859

Proportional hazards survival analysis (Likelihood-ratio tests presented here, *n *= 82) of the effects of water availability, molecular form, body size, and presence of oocysts and/or sporozoites on the survival (time of death) of blood-fed *An. gambiae *s.s. female mosquitoes collected from huts in an area endemic for malaria and brought to the laboratory. Females were kept for seven days with water *ad-libitum *or with restricted access to water. Interactions were non-significant.

**Figure 2:**
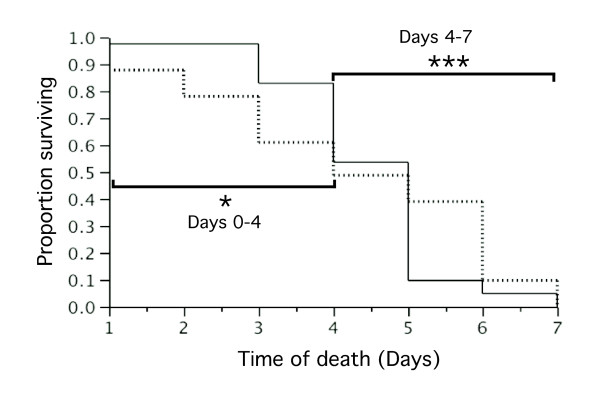
**Survival of *An. gambiae *females with constant daily access to water (solid line) or access to water restricted to 16 h per day (dashed line) during the 7-day hydric stress experiment**. Separate statistical tests were conducted on female survival during the first (day 0 - mid-day 4) and second (mid-day 4 to day 7) halves of the experiment revealing a strong interaction between the stage of the experiment and the effect of hydric stress. Level of significance are *P *< 0.05 *, *P *< 0.005 **, *P *< 0.001 ***.

In addition to analysing overall effects on female survival during the 7-day stress experiment (see above), separate analyses of the effect of hydric stress for early (day 0 to 4) and late stage of the 7-day stress experiment (day 4 to 7) were conducted in order to detect potential parasite-induced mortality in early infection stages (Table 1, hypothesis 3). The 82 females that died during the 7-day hydric stress period (41 stressed and 41 non-stressed females) were split between those that died in the first 4 days of the hydric stress period (*n *= 31) and those that died later (*n *= 51). Stressed females (*n *= 19) died significantly faster than non-stressed females (*n *= 12) during the first 4 days of the hydric stress period thereby following a pattern of mortality compatible with that of increased parasite-induced mortality in combination with stress (Wilcoxon: *n *= 31, *df *= 1, *χ*^2 ^= 4.6, *P *= 0.032)(Figure 2). The reverse pattern of mortality was observed during the second half of the 7-day hydric stress period (Wilcoxon: *n *= 51, *df *= 1, *χ*^2 ^= 12.6, *P *< 0.001)(Figure. 2) supporting the idea that late-dying (day 4-7) mosquitoes in the low water-availability group were individuals of higher phenotypic quality that generally better resisted both infection and stress.

Overall survival of females during the desiccation challenge was independent of water availability during the seven preceding days and of the presence of sporozoites (Figure 1d). However it was significantly negatively affected by oocyst presence (Figure 1b), the molecular form of females and their body size (Table 6). The six females carrying oocysts survived on average 11.0 h (8.7-13.3CI) compared to 15.1 h (14.7-15.6CI) for non-infected ones, which represents an average 27.2% reduction in survival. Despite highly unbalanced numbers of oocyst-infected and non-infected females, the large sample and effect sizes made for very high statistical power to detect survival differences between the two groups (Wilcoxon: *n *= 318, *χ*^2 ^= 14.6, *P *< 0.001, power= 99%). Females of the M molecular form survived on average 15.5 h (14.9-16.1CI) or 9.1% longer than the average 14.2 h (13.7-14.8CI) of S-forms. Finally, survival significantly positively correlated with female body size.

**Table 6:** Analysis of determinants of female survival during the desiccation challenge

Source	*df*	L-R *Chi*-square	*p*-value
Water availability	1	0.85	0.356
Molecular form	1	9.63	**0.002**
Body size	1	3.87	**0.049**
Oocyst(s) presence	1	7.59	**0.006**
Sporozoites presence	1	0.01	0.924

Proportional hazards survival analysis (Likelihood-ratio tests presented here, *n *= 318) of the effects of water availability, molecular form, body size, and presence of oocysts and/or sporozoites on the survival (time of death) of blood-fed *An. gambiae *s.s. female mosquitoes collected from huts in an area endemic for malaria and brought to the laboratory. Females were subjected to continuous desiccation till death following a 7-day period either with water *ad libitum *or with restricted access to water. Significant *p*-values are in bold. Interactions were non-significant.

## Discussion

The results of this study suggest indirectly and directly that *An. gambiae *s.s. females incur fitness costs in terms of reduced survival when infected with oocysts of the human malaria parasite *P. falciparum *and submitted to hydric stress. This is the first study reporting a potential decrease in female survival associated with *Plasmodium *infection in naturally-infected females.

Over the course of the entire experiment, significantly lower numbers of oocyst carriers amongst females that had limited access to water were found. This result suggests that a higher proportion of females exposed to hydric stress might have died early in the course of infection at a stage where ookinetes or young oocysts could not be detected by microscopy. Because contaminant DNA from the bloodmeal complicates the detection of ookinetes and very young oocysts by PCR, the interactive effects of hydric stress and *Plasmodium *infection on female survival could not be directly assessed and was indirectly corroborated by comparing survival curves of stressed and non-stressed females. Whilst a direct assessment of ookinete and young oocysts-induced mortality would have been ideal, distinguishing mosquitoes that die from direct parasite effects, the costs of mounting an immune response, the direct effects of hydric stress, or combinations of these factors will always constitute a difficult challenge. From a technical point of view, separating the bloodmeal for PCR-based analysis whilst keeping the midgut intact for screening under the microscope is particularly challenging and very time-consuming and no practical ways to circumvent this problem were found. Detecting older oocyst infections from previous bloodmeal in order to differentiate their potential effects from those of potential new infection was clearly the first priority. Thus mid-gut dissections, whilst not allowing for detecting very young infections, were the best compromise between these different contingencies.

One could argue that a lower prevalence of oocyst in the stressed group could also be explained if hydric stress would prevent the development of ookinetes and young oocysts prevalence. However, that interpretation was not deemed plausible as it would go against the long-established principle that parasites are costlier to their host when these face harsher conditions or limited resources [34-36]. It would also suggest an altruistic-like behaviour of the parasite, which would itself die whilst leaving its host with enough resources to survive stress and infection. Finally it does not satisfactorily explain why the lower oocyst prevalence found in stressed females would be accompanied by an early peak of female mortality as discussed in the next paragraph.

As predicted if fitness costs due to parasite infection occurred mostly during the early stages of infections and were exacerbated when females were simultaneously stressed, female survival decreased significantly during the first days following their bloodmeal when subjected to hydric stress. It is noteworthy that these patterns of female survival and infection are distinct from those predicted for other possible hypotheses (Table 1). It seems unlikely that hydric stress alone could have led to such contrasted pattern of mortality between stressed and non-stressed females during the first and second half of the 7-day stress experiment (Table 1, hypothesis 2). This is because preliminary experiments were conducted in order to find an optimal level of stress that would not result in differential mortality (and result in differential phenotypic quality). In 2 independent experiments with data on mortality of 113 and 283 females from a colony of the Mopti form, the 16 h water availability regime did not lead to significant differences between experimental groups (*p *> 0.4 in both cases) nor in such contrasted patterns between early mortality and that at later stages. In the experiment presented here, hydric stress did not significantly increase the mortality in stressed females, instead it strongly altered the shape of their survival curve as compared to that of non-stressed females. Thus the data does not provide evidence for a direct effect of hydric stress combined or not with early or late parasite-induced mortality (Table 1, hypotheses 2, 4 and 6 respectively). Nor does it support the idea that mortality would solely be induced by infection and occur at a more advanced stage (day4-7) of oocysts development (Table 1, hypothesis 5). Oocysts at that stage are easily detected by dissection and staining and no direct impact on survival was detected (Figure 1a). The fact that surviving stressed females survived significantly better than non-stressed ones from day 4 to 7 supports the assumption that natural parasite infections do not occur at random but affect less resistant phenotypes more than others. This assumption is thought to be one of the main explanations for the negative binomial parasite distribution amongst hosts observed in many parasitic infections including Plasmodium-infected mosquitoes [7,37]. It may also be that early-dying individuals were on average older mosquitoes and that age maybe yet another factor interacting with stress and infection to determine fitness costs. Because a large number of field-collected females were randomly allocated to each of two experimental groups, comparable age structure and proportion of females that had just fed on an infected blood meal were expected in both groups. Despite that, developing methods that would also enable to age females in simpler and more accurate ways than current methods based on ovarian morphology [38], would have allowed to add age as a covariate in the analyses and would greatly facilitate the interpretations of future studies based on naturally-infected females. To identify those individuals that were exposed to an infective blood meal without developing an infection would also be extremely interesting but it is simply not feasible in the case of natural infection.

The greatly reduced survival of oocyst-carrying females facing a sustained desiccation period suggests that negative fitness effects could also be associated with later stages of oocyst development (6 days and older). Significant differences in the mean survival of infected and non-infected females could be detected despite the relatively low prevalence of natural oocyst infections and the vulnerability of newly infected females because the effect on mean survival was so drastic and the sample size, although imbalanced, was very large. Although it could be argued that females might not often face desiccation challenges of that intensity in the wild, there is ample evidence that drought-imposed selection on mosquito populations determine their distribution and abundance (see section 1). Very few individuals survive seasonal droughts and estivate to the next rainy season suggesting that desiccation stress and desiccation-induced mortality may not be uncommon events in sub-Saharan mosquito populations.

The manipulation of water availability in the first days of the experiment constituted a much more subtle level of stress which only contributed to mortality in combination with infection. The results seem to confirm the suggestion that insectary condition may not be adequate for revealing *Plasmodium*-induced fitness costs because these may only be expressed under natural environmental conditions where a variety of environmental stressors are present [12]. This could provide a simple explanation for the overall lack of parasite-induced costs reported from laboratory studies of natural *Anopheles/Plasmodium *interactions [5].

In contrast to what was observed for oocyst infections, no evidence of sporozoite-induced mortality was found in the course of the experiment. Sporozoites prevalence measured during the 7-day stress period, where lower than those based on mosquitoes surviving to the desiccation challenge. This pattern can be expected because sporozoites developed from older infections that appear to have continued their development to the sporozoite stage without leading to the mortality observed in newly-infected and stressed females. The resulting larger sample sizes of sporozoite-infected females did not reveal significant effects of the 7-day hydric stress period or of the desiccation challenge on survival, suggesting that if negative effects do occur they are of small biological importance. These results are in line with those of Chege and Beier [14], who failed to find any direct effect of sporozoite infections on the survival of *An. gambiae *and *Anopheles funestus*. Sporozoite-infected mosquitoes are however known to indirectly incur higher mortality through host behavioural defences because of increased probing and feeding behaviour [15,39].

There are several potential mechanisms that could explain the fitness costs of ookinetes and developing oocysts in combination to hydric stress. Ookinetes, being extremely small, probably divert little resource directly from their hosts. However, they perforate the midgut wall in order to reach the other side of the basal lamina before developing into oocysts. This physical damage could interfere with water balance regulation and cause mortality, particularly in hydric-stressed females. The ingestion of infected blood is also known to trigger a powerful immune response [40-42], which peaks 24-48 hours post feeding and is maintained throughout the infection albeit at a lower level [43,44]. That immune reaction burst coincides with a drastic reduction in the number of invading parasites [43,45]. Immune reactions also carry an energetic cost which is thought to reduce host fecundity through trade-offs between immunity and reproduction [46-48]. Thus the host immune response, and particularly its initial peak which is designed to clear the infection at an early stage, could also carry costs in terms of survival if trade-offs exist between immune response mechanisms and physiological adaptations for resisting desiccation.

As oocyst develop, they may divert larger amounts of resources from the host for their own growth, leading to direct energetic trade-offs with body condition which is an important determinant of desiccation resistance in Dipterans [23,49]. The correlation found between survival to the desiccation challenge and body size supports the idea that larger mosquitoes are better able to cope with desiccation stress, either through better body condition or because of the allometric relationship between body size and transpirational water loss [49-51]. If trade-offs exist between the immune response and mechanisms of desiccation resistance, then these may continue to negatively affect the desiccation resistance of infected females as oocysts develop. Generally, the patterns of early mortality observed in this study suggests that the ookinetes and/or immature oocysts stages represent critical stages in terms of mortality but that oocysts continue being costly later in their development. In this context, the lack of survival costs associated with sporozoites presence could be due to the fact that the sporozoite stage is not associated with an increase in parasite biomass and might not divert much resource from its host. This may, in turn, diminish the need for a strong host immune response, which would decrease the overall costs of being infected with sporozoites even further. Sporozoites do migrate through the wall of the salivary glands and, as such, could cause damaging effects to mosquitoes but the results reported here and those of previous studies do not suggest that this translates directly into increased mortality [14].

No evidence of differences in susceptibility to infection between the M Mopti females (five oocysts and 25 sporozoite-positive females), and females of the S Bamako and Savanna chromosomal forms (eight oocysts and 25 sporozoite-positive females) was found, a result which is consistent with the results of previous but unpublished comparisons of vector competence between the two molecular forms in Mali (Sékou Traoré Pers. comm.) and with a comparison of infection prevalence between populations of the two molecular forms in Cameroon [52]. As expected from the positive correlation observed between rainfall and the abundance of chromosomal arrangements typical of the Mopti form in field populations, we found that females of the M Mopti form survived longer when faced with constant desiccation [19,20]. Similar patterns have recently been reported by Lee *et al *[21], who used the progeny of field-collected females to compare the form's desiccation resistance. Although no significant interaction between the effects of infection and molecular form on survival was found, this result should be taken with caution given the overall low number of females infected with oocysts, which gave particularly low power to detect such interaction. Thus, the possibility of an interaction between the stress of infection and that of desiccation cannot fully be dismissed at this stage and will require further testing.

## Competing interests

The authors declare that they have no competing interests.

## Authors' contributions

FT and FAA planned and designed the experiment with inputs from MC and HH, FAA conducted the experiments with AG, AST, and FT. FT and FAA conducted the data analyses and wrote the manuscript. All authors read and approved the final manuscript.
